# Correcting the Activity-Specific Component of Heart Rate Variability Using Dynamic Body Acceleration Under Free-Moving Conditions

**DOI:** 10.3389/fphys.2018.01063

**Published:** 2018-08-07

**Authors:** Kazato Oishi, Yukiko Himeno, Masafumi Miwa, Hiroki Anzai, Kaho Kitajima, Yudai Yasunaka, Hajime Kumagai, Seiji Ieiri, Hiroyuki Hirooka

**Affiliations:** ^1^Laboratory of Animal Husbandry Resources, Division of Applied Biosciences, Graduate School of Agriculture, Kyoto University, Kyoto, Japan; ^2^Department of Bioinformatics, College of Life Sciences, Ritsumeikan University, Shiga, Japan; ^3^Division of Grassland Farming, Institute of Livestock and Grassland Science, National Agriculture and Food Research Organization (NARO), Tochigi, Japan; ^4^Department of Animal and Grassland Sciences, Faculty of Agriculture, University of Miyazaki, Miyazaki, Japan

**Keywords:** dynamic body acceleration, free-moving condition, heart rate correction, heart rate variability, physical activity

## Abstract

Heart rate variability (HRV) analysis is a widely used technique to assess sympatho-vagal regulation in response to various internal or external stressors. However, HRV measurements under free-moving conditions are highly susceptible to subjects’ physical activity levels because physical activity alters energy metabolism, which inevitably modulates the cardiorespiratory system and thereby changes the sympatho-vagal balance, regardless of stressors. Thus, researchers must simultaneously quantify the effect of physical activity on HRV to reliably assess sympatho-vagal balance under free-moving conditions. In the present study, dynamic body acceleration (DBA), which was developed in the field of animal ecology as a quantitative proxy for activity-specific energy expenditure, was used as a factor to correct for physical activity when evaluating HRV in freely moving subjects. Body acceleration and heart inter-beat intervals were simultaneously measured in cattle and sheep, and the vectorial DBA and HRV parameters were evaluated at 5-min intervals. Next, the effects of DBA on the HRV parameters were statistically analyzed. The heart rate (HR) and most of the HRV parameters were affected by DBA in both animal species, and the inclusion of the effect of DBA in the HRV analysis greatly influenced the frequency domain and nonlinear HRV parameters. By removing the effect of physical activity quantified using DBA, we could fairly compare the stress levels of animals with different physical activity levels under different management conditions. Moreover, we analyzed and compared the HRV parameters before and after correcting for the mean HR, with and without inclusion of DBA. The results were somewhat unexpected, as the effect of DBA was a highly significant source of HRV also in parameters corrected for mean HR. In conclusion, the inclusion of DBA as a physical activity index is a simple and useful method for correcting the activity-specific component of HRV under free-moving conditions.

## Introduction

Heart rate variability (HRV) is an effective indicator of the activities of the autonomic nervous system (i.e., the balance between sympathetic and vagal activity) and is widely used to assess the autonomic response to various internal and external factors (Acharya et al., 2006; von Borell et al., 2007; Billman, 2011). Indeed, the responses to a variety of physiological, psychological, and clinical factors such as exercise effects (Pradhapan et al., 2014; Valenzano et al., 2016), anxiety (Chalmers et al., 2014; Cotoia et al., 2018), and health conditions (e.g., cardiac diseases, Watanabe et al., 2017; Ciliberti et al., 2018; Sessa et al., 2018; viral infection, Carter et al., 2014; and obesity, Mehta, 2015; Messina et al., 2017; Triggiani et al., 2017) have been intensively investigated using HRV analysis in recent human studies. However, HRV is simultaneously affected by several interdependent physiological and environmental factors (Fatisson et al., 2016) because the sinus node acts as the final summing element of stimuli from the sympathetic and vagal nerves, and their relationship is reflected in the actual heart inter-beat intervals (Voss et al., 2009). Moreover, HRV is strongly influenced by the change in average heart rate (HR) due to the simple mathematical problem of the inverse non-linear relationship between HR and inter-beat intervals (Sacha and Pluta, 2008; Billman, 2013a). The same changes in HR cause much greater fluctuations of inter-beat intervals for the slower average HR than for the faster one (Sacha, 2013). Therefore, investigations of HRV should ensure a similar environment for each subject to avoid the effect of changes in HR on HRV (Task Force of the European Society of Cardiology, North American Society of Pacing Electrophysiology, 1996). However, the environment of the subjects undergoing HRV assessments using ambulatory monitoring systems under freely moving conditions is virtually impossible to standardize. Nevertheless, there is a strong need to assess HRV under free-moving conditions in 24-h health monitoring contexts or in several HRV studies, such as psychophysiological studies.

Several methods for correcting the HRV for HR have been proposed to differentiate between the HRV changes that are directly related to the cardiac autonomic response and those changes that are mathematically related to the baseline HR (Sacha et al., 2013a,b; Monfredi et al., 2014; Billman et al., 2015; Estévez-Báez et al., 2015; Bolea et al., 2016). By suitably modifying the relationship between HRV and HR, researchers have increased the analytical value of HRV (Sacha, 2014). However, although the relation between HRV and HR has been shown to be partially due to mathematical and biophysical properties (Zaza and Lombardi, 2001; Monfredi et al., 2014), part of this association is also due to the concurrent influence of the autonomic nervous system on both parameters, and consequently, corrections for HR might mask differences in HRV (Herzig et al., 2017). The influence of other factors that promote variability should not be dismissed; thus, the best approach for disentangling the interdependence of mean HR and HRV is still being debated (Silva et al., 2017).

Since HR displays a strong relationship with physical activity intensity across different types of activity (Brage et al., 2007), a change in physical activity levels is thought to be one of the major modulators of HRV if these changes occur in the period of analysis (Bernardi et al., 1996). Hence, to understand the interrelationships between physiological and/or environmental factors and HRV under free-moving conditions, we expect to remove the confounding effect of physical activity directly from the evaluation of HRV. At present, physical activity and HRV have been simultaneously measured in several studies with different aims; some studies collected accelerometer data only to delete the sections of the heart inter-beat interval data with excessive movement before the HRV analysis (Hansen et al., 2003; Johnsen et al., 2003; Verkuil et al., 2016), and others examined the relationships between HRV and physical activity (Grossman et al., 2004; Hautala et al., 2010; Taniguchi et al., 2015; Tonello et al., 2016; Herzig et al., 2017). Although the latter studies revealed substantial effects of physical activity on HRV indices, a general consensus on the strategy for separating the influence of physical activity on HRV from the effects of other regulatory processes is still unavailable (Laborde et al., 2017).

Among several qualitative or quantitative physical activity indices, acceleration is highly sensitive to subjects’ movement levels (Willener et al., 2015). In particular, an acceleration index called dynamic body acceleration (DBA) is used in the field of wild animal ecology to quantify the three-dimensional movement of animals with accelerometers (Wilson et al., 2006). This index is calculated by extracting static accelerations (due to gravity) from raw three-dimensional accelerations, because the dynamic parts of acceleration signals are strongly related to the animal’s movement (Halsey et al., 2011). In addition, robust relationships between DBA and the rate of oxygen consumption have been reported in a wide range of bipedal and quadrupedal species (several bird species and mammalian species, including humans) (Halsey et al., 2009b, 2011; Miwa et al., 2015), and therefore several studies have used DBA in field experiments to evaluate animals’ activity-specific energy use (e.g., Shepard et al., 2009; Wilson et al., 2012). Since HRV is greatly affected by the change in respiration to meet the energy requirement caused by physical activity (Grossman and Taylor, 2007), the use of DBA as a quantified physical activity index appears to be a promising method for correcting the activity-specific component of HRV under free-moving conditions.

The objective of the present study was to examine the possibility of utilizing DBA as a correcting factor in the analysis of HRV. First, the relationship between DBA and HRV was analyzed, and then, the effect of DBA on HRV was included in the HRV analysis to correct the effect of physical activity on HRV. To simplify the effects of other factors (e.g., type and nature of physical activity and of emotional circumstances) on HRV as much as possible under daily living conditions, farm animals (cattle and sheep) were used as representative subjects in the present study. The HRV of the animals was assessed under two management systems: a housing system (periodic indoor feeding with relatively lower activity) and a grazing system (freely grazing in a pasture with relatively higher activity); the stress levels were compared fairly between the systems by correcting the effect of physical activity on HRV using DBA. To the best of our knowledge, this is the first study to assess the autonomic balance by correcting the effect of quantitative activity levels on HRV using DBA evaluated via accelerometry.

## Materials and Methods

### Study Sites, Periods and Tested Animals

Eleven animals from two domestic animal species (cattle as large animals and sheep as small animals) were used as model animals to assess HRV under freely moving conditions in the present study (**Table 1**). None of the tested animals had health problems, and the mean ambient temperature at all experimental sites appeared to be within the thermoneutral zone for the animals, according to the Commonwealth Scientific and Industrial Research Organisation [CSIRO] (2007).

**Table 1:** Characteristics of tested animals in the present study.

	Cattle	Sheep
*n*	7	4
Breed	Japanese Brown	Corriedale
Sex	Cow	Cast rated
Body weight (kg)	589.7 ± 45.4	42.8 ± 4.5
Age (months)	124.0 ± 42.4	47.5 ± 0.3

*The experiments with cattle were conducted at Kumamoto Prefectural Farm (Aso), Japan, in September 2014 (three animals) and August 2015 (four animals). The experiments with sheep were conducted at Kyoto University, Japan, in January and February 2015.*

In the present study, data were collected from the tested animals under two management systems (housing and grazing) used for the same individuals. Data were collected from animals under these two management systems over two consecutive days after adaptation. All tested animals were well adapted to both the housing and grazing management systems before experiments. The areas allocated for the housing and grazing management systems were wide enough for the animals. The animals within the housing management system were provided a sufficient amount of feed to maintain their weight twice a day (9:00 and 17:00) and had free access to water. The animals within the grazing management system had free access to pastures and water and were given supplemental feed, if necessary, to maintain their weight. Detailed information about the pasture conditions and the feeding management systems is presented in our previous report (Miwa et al., 2017).

This study was carried out in accordance with the recommendations of the Guideline of the Animal Experiment Committees of Kyoto University. All animal experiments were approved by the Animal Experiment Committees of Kyoto University (Permit Numbers: 26–76) and were permitted by the farm manager at the Kumamoto Prefectural Farm. All procedures for equipping the animals with data loggers were performed as quickly as possible to minimize the animals’ discomfort.

### Measurements of Body Acceleration and Inter-Beat Intervals

Simultaneous measurements of the body acceleration and cardiac inter-beat intervals of the tested animals was executed according to the method proposed by Miwa et al. (2015). Acceleration was measured using acceleration data loggers (USB Accelerometer X6-1A, X6-2, Gulf Coast Data Concepts, Waveland, MS, United States) that were set to separately record the acceleration (±2 *g*) in three axes at 10 Hz with 16-bit resolution. Because DBA is designed to measure acceleration around an animal’s center of mass (Halsey et al., 2011), the acceleration logger was attached on the top of each animal’s back (behind the withers). The animals’ inter-beat intervals were measured using a heart rate monitor (Polar Electro Heart Rate Monitor RS800CX, Polar Electro, Kempele, Finland). Although the waveform of the electrocardiogram of ungulates, particularly the QRS complex, is different from that of humans (Sawazaki et al., 1976), the heart rate monitors have been widely used to measure inter-beat intervals with guaranteed success not only in humans but also in several animal species, including ungulates such as cattle, goats, sheep, pigs, and horses (e.g., von Borell et al., 2007; Essner et al., 2013; Younes et al., 2016). Electrodes were placed on the animal’s right shoulder and left anterior thorax and fixed with a homemade belt. The electrodes were connected to the transmitter of the heart rate monitor with leads for extension. With infrared connections to the transmitter, the heart rate monitor recorded the inter-beat interval data during the experiment. At the end of the entire experimental period, all loggers were immediately removed, and the data were collected.

### Data Processing and Calculation

**FIGURE 1:**
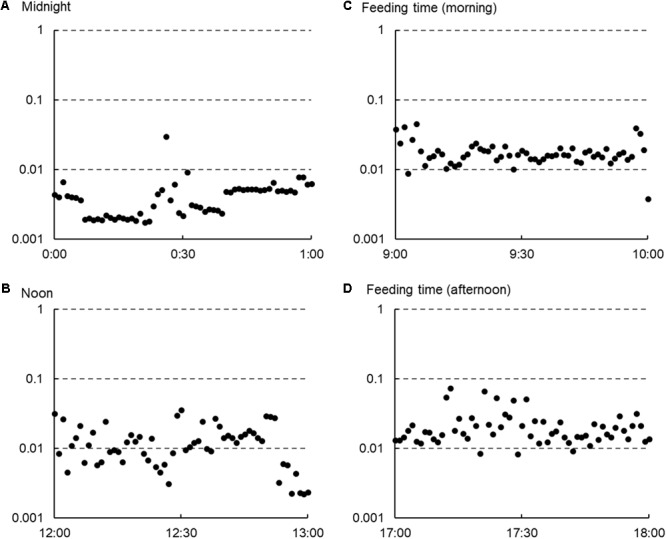
Representative changes in the average raw vectorial dynamic body acceleration (VeDBA: *g*) values per minute observed in a cow managed under housing conditions within 1 day. The horizontal axes show the time of day, and the vertical axes show the common logarithmic scale for the raw VeDBA values. The values tended to be stable and lower at midnight **(A)**, varied and higher at noon **(B)**, and much higher during feeding times **(C,D)**.

The three-dimensional acceleration data were converted from digital counts into the standard acceleration of gravity (*g*). The raw acceleration values in each axis resulted from the combination of static acceleration (due to gravity) and dynamic acceleration (due to movement). In the present study, the vectorial sum of DBA in three dimensions (vectorial DBA, VeDBA; Qasem et al., 2012) was used as a quantified physical activity index related to activity-specific energy expenditure and was derived using the procedure listed below. (i) Static acceleration was first approximated by smoothing the obtained acceleration using 2-s running means, according to the method described by Halsey et al. (2009b). (ii) Dynamic acceleration was calculated by subtracting the static acceleration from the raw acceleration. (iii) The vectorial sum of the derived dynamic accelerations across three orthogonal axes was calculated as VeDBA: VeDBA = (DA*x*^2^ + DA*y*^2^ + DA*z*^2^)^0.5^, where DA*_x_*, DA*_y_*, and DA*_z_* represent dynamic acceleration in each axis (*g*). **Figure 1** shows the representative VeDBA data that were collected from an animal in four different periods within 1 day. VeDBA values were transformed into a natural logarithm for normalization, and the averaged values of the natural logarithmically transformed VeDBA over 5 min (for synchronizing with HRV data) were used for the analysis of the relationship between VeDBA and HRV parameters.

Data on inter-beat intervals were derived from the loggers using Polar Pro Trainer 5 software (Polar Electro, Kempele, Finland). The recorded inter-beat intervals were used to calculate HRV parameters using Kubios 2.2 software (Biomedical Signal Analysis Group, Department of Applied Physics, University of Kuopio, Finland: Tarvainen et al., 2014). The inter-beat intervals for each animal were divided into 5-min interval series, as recommended by the Task Force for the analysis of HRV (Task Force of the European Society of Cardiology, North American Society of Pacing Electrophysiology, 1996), after artifact correction using the custom filter of the software. The 5-min inter-beat interval data with values outside of the 3-sigma range for each dataset were removed as outliers to increase the accuracy of the analysis. Although a universal method for editing inter-beat interval data is not available, the general consensus is that artifacts should be deleted before performing the analyses (Task Force of the European Society of Cardiology, North American Society of Pacing Electrophysiology, 1996). The proportion of data removed by the procedure was 0.66%.

For each 5-min interval, several HRV parameters were calculated using four different analyses: time domain analysis, frequency domain analysis, Poincaré measures and the recurrence quantification analysis (RQA). For the parameters in the time domain analysis, in addition to the mean HR, the root mean square of the successive inter-beat interval differences (RMSSD) was calculated. In the frequency domain analysis, the normalized power of the high frequency band (HF) and the ratio of the normalized power of the low frequency band to HF (LF/HF) were calculated using the Fast Fourier Transformation of the power spectrum analysis. The LF ranges were set to 0.05–0.20 Hz for all animals, and the HF ranges were set to 0.20–0.58 Hz for cattle and 0.20–0.40 Hz for sheep, as described by von Borell et al. (2007). The Poincaré plot is a graphical representation of the relationship between inter-beat intervals and the successive inter-beat intervals, and the plot is fitted to an ellipse. The Poincaré measures used to quantify HRV are geometric parameters that are derived from the diameters of the ellipse; the diameter perpendicular to *y* = *x* represents standard deviation 1 (SD1) and indicates the short-term variability of HRV, and the other diameter represents standard deviation 2 (SD2) that indicates the long-term variability of HRV. In the present study, SD1 and the ratio of SD2 to SD1 (SD2/SD1) were evaluated as Poincaré measures. To evaluate nonlinear complex processes involved in HRV, RQA was used in the present study. The recurrence plot method proposed by Eckmann et al. (1987) is also a graphical tool used to measure the time constancy of dynamic systems, and several RQA measures used to quantify recurrence plots of heart inter-beat intervals were developed by Zbilut et al. (2002). The length of the longest line of recurrent points (*L*_max_) and the percentage of recurrent points forming diagonal lines (%DET) were calculated from these measures for subsequent analyses. In general, animals with low HRVs display high sympathetic activity and can be associated with an increased risk of “stress”; RMSSD, HF, and SD1 are positively related with HRV, whereas other parameters measured in the present study are negatively related with HRV. For a more detailed description of the HRV measurements, please see several reviews, such as those by Acharya et al. (2006), von Borell et al. (2007), and Billman (2011).

The 5-min interval datasets that included both VeDBA and HRV parameters were analyzed. A total of 9,549 data points, an average of 434.0 data points (35.8 h) per animal under each management system, were used for the analysis.

### Statistical Analysis

We first estimated the correlation coefficients between VeDBA and the mean HR and those between VeDBA and the HRV parameters for each animal to evaluate the relationship between physical activity levels quantified by calculating VeDBA and HRV; then, we averaged the coefficients for each animal species. After investigating the correlations, the effects of treatments on the mean HR and the HRV parameters were analyzed for each animal species by correcting the effect of VeDBA on the mean HR and the HRV parameters using the following mixed linear model:

(1) $$\begin{array}{c} Y_{\mathrm{ijk}}\;=\;\mu \;+\;T_{i}\;+\;A_{j}\;+\;\beta {\left(\mathrm{VeDBA}\right)}_{\mathrm{ijk}}\;+\;e_{\mathrm{ijk}} \end{array}$$

Where, *Y*_ijk_ is the value of the mean HR or the HRV parameters in 5 min, *μ* is the overall mean, *T*_i_ is the fixed effect of treatments (the difference in management systems, housing vs. grazing), *A*_j_ is the random effect of individuals, *β*(VeDBA)_ijk_ is the covariate of VeDBA, and *e*_ijk_ is the residual error. This statistical model, which includes the effect of physical activity quantified using DBA, can be applied to evaluate the effects of variable factors that are considered relevant to stress or pathological conditions on HRV parameters as fixed effects in various HRV investigations under free-moving conditions, and in the present study, the difference in management systems was treated as the fixed effect. The model excluding the covariate of VeDBA was also evaluated to analyze the influence of including VeDBA in the model, and the results obtained from the two models were compared. In addition, because daily activities such as feeding and sleeping could affect DBA and HRV, the relationship between the diurnal changes in DBA and HRV was also analyzed in the present study. The model adding the fixed effect of time of day (eight periods per day) and the interaction effect between the time of day and the management systems in the statistical model (1) was analyzed to evaluate this relationship in animals under the two management systems. The differences in all of the analyses were analyzed using the least squares means with the Tukey–Kramer adjustment and were considered significant at *P* < 0.05. The correlation coefficient was calculated with PROC CORR, and other analyses were performed by PROC MIXED in SAS 9.3 (SAS Institute, 2008).

## Results

**Table 2** shows the means and standard deviations of the variables obtained in the present study. Although most of the values largely varied between animal species and even among individual animals, the arithmetic means of VeDBA, mean HR, LF/HF, and the two RQA parameters were lower for animals under the housing conditions than for animals under the grazing conditions.

**Table 2:** Mean and standard deviations of VeDBA, mean HR, and HRV parameters in animals under the two management systems.

	Cattle (*n* = 7)		Sheep (*n* = 4)	
	Housing	Grazing	Housing	Grazing
VeDBA	–4.90 ± 0.86	–4.34 ± 1.08	–5.24 ± 1.13	–4.16 ± 1.16
Time domain				
Mean HR	59.11 ± 12.37	68.68 ± 19.72	103.80 ± 38.94	112.69 ± 30.23
RMSSD	29.73 ± 17.44	29.94 ± 23.76	81.74 ± 29.60	63.07 ± 29.44
Frequency domain				
HF	20.00 ± 15.00	21.50 ± 16.85	37.02 ± 18.63	33.70 ± 19.58
LF/HF	10.05 ± 14.79	11.03 ± 16.11	2.96 ± 3.69	3.71 ± 3.97
Poincaré measures				
SD1	21.07 ± 12.36	21.21 ± 16.83	57.87 ± 20.96	44.65 ± 20.84
SD2/SD1	5.34 ± 2.80	5.59 ± 3.86	2.90 ± 3.91	2.61 ± 1.31
RQA				
*L*_max_	225.38 ± 90.92	239.95 ± 108.45	174.63 ± 142.38	206.90 ± 148.33
%DET	99.33 ± 1.11	99.38 ± 0.98	97.93 ± 1.98	98.26 ± 1.95

*Values are presented as the mean and standard deviations of the coefficients for the animals. Mean HR (beats/min) and HRV parameters were calculated for each 5-min interval. VeDBA, natural logarithmically transformed vectorial dynamic body acceleration (*g*); HR, heart rate; HRV, heart rate variability; RQA, recurrence quantification analysis; RMSSD, root mean square of the successive inter-beat intervals differences (ms); HF, normalized power of the high frequency band (n.u.); LF/HF, the ratio of normalized power of the low frequency band (LF) to HF; SD1, standard deviation of instantaneous HRV measured from axis 1 in Poincaré plot (ms); SD2/SD1, the ratio of standard deviation of long-term HRV measured from axis 2 of Poincaré plot to SD1; *L*_*max*_, the length of longest line of recurrent points (beats); %DET, percentage of recurrent points forming diagonal lines.*

The correlation coefficients between VeDBA and the mean HR and those between VeDBA and the HRV parameters for each animal species are shown in **Table 3**. Positive correlations with the mean HR, LF/HF, SD2/SD1, and the two RQA parameters and negative correlations with the other parameters were observed in both animal species, indicating that the physical activity quantified by VeDBA affected the mean HR and the HRV parameters in a manner similar to a stressor. Despite the large magnitude of differences in the coefficients among individual animals in each species, the averages of the absolute values of the coefficients were 0.363 for cattle and 0.404 for sheep, indicating that the HRV parameters chosen in the present study were more or less correlated with VeDBA. The coefficients obtained as absolute values differed, even between 2 parameters chosen within the same type of analysis, but the differences were smaller for parameters in the frequency domain and RQA analyses than for parameters in the other analyses. When the coefficients were compared between species, large differences were observed in time domain parameters and Poincaré measures, whereas relatively smaller differences were observed in other parameters, particularly RQA parameters.

**Table 3:** Correlation coefficients between VeDBA and mean HR and between VeDBA and HRV parameters.

**Cattle**		**Sheep**	
Time domain		Time domain	
Mean HR	0.7014 ± 0.2247	Mean HR	0.3046 ± 0.4977
RMSSD	–0.1945 ± 0.3775	RMSSD	–0.5081 ± 0.0704
Frequency domain		Frequency domain	
HF	–0.3229 ± 0.2581	HF	–0.5214 ± 0.1101
LF/HF	0.3294 ± 0.1967	LF/HF	0.3722 ± 0.1222
Poincaré measures		Poincaré measures	
SD1	–0.1948 ± 0.3774	SD1	–0.5082 ± 0.0702
SD2/SD1	0.3729 ± 0.2535	SD2/SD1	0.2270 ± 0.2092
RQA		RQA	
*L*_max_	0.4522 ± 0.2589	*L*_max_	0.4036 ± 0.0201
%DET	0.3368 ± 0.1517	%DET	0.3842 ± 0.1131

*Values are presented as the mean and standard deviations of the coefficients for individual animals. VeDBA, natural logarithmically transformed vectorial dynamic body acceleration (*g*); HR, heart rate; HRV, heart rate variability; RMSSD, root mean square of the successive inter-beat intervals differences (ms); HF, normalized power of the high frequency band (n.u.); LF/HF, the ratio of normalized power of the low frequency band (LF) to HF; SD1, standard deviation of instantaneous HRV measured from axis 1 in Poincaré plot (ms); SD2/SD1, the ratio of standard deviation of long-term HRV measured from axis 2 of Poincaré plot to SD1; RQA, recurrence quantification analysis; *L*_*max*_, the length of longest line of recurrent points (beats); %DET, percentage of recurrent points forming diagonal lines.*

**Table 4** shows the effects of including VeDBA as a covariate on the mean HR and the HRV parameters within the two management systems. When VeDBA was not considered in the model, mean HR, LF/HF, and *L*_max_ were significantly higher under grazing conditions than under housing conditions in both animal species (*P* < 0.05), indicating a higher sympathetic activity in animals managed under grazing conditions than those managed under housing conditions. However, when VeDBA was used as a covariate in the analysis, the effect of VeDBA was a highly significant source of variation in all parameters (*P* < 0.001), and most of the values for parameters obtained from the frequency domain analysis and RQA showed significant opposite trends compared with the results obtained without including the effect of VeDBA (*P* < 0.05). The inclusion of the effect of VeDBA strongly affected the parameters of the frequency domain analysis and RQA but did not affect the results of the other two analyses.

**Table 4:** The effects of including VeDBA as a covariate on mean HR and HRV parameters in animals under the two management systems.

	Without the effect of VeDBA		With VeDBA as a covariate	
	Housing	Grazing	Housing	Grazing
Cattle				
Time domain				
Mean HR	56.95 ± 4.09a	67.17 ± 4.09b	58.57 ± 3.72c	65.16 ± 3.72d
RMSSD	30.02 ± 3.50a	31.15 ± 3.51b	29.60 ± 3.47c	31.66 ± 3.48d
Frequency domain				
HF	20.25 ± 1.90a	21.93 ± 1.91b	19.00 ± 1.81c	23.48 ± 1.82d
LF/HF	9.53 ± 2.15a	10.61 ± 2.16b	10.65 ± 2.03d	9.22 ± 2.04c
Poincaré measures				
SD1	21.27 ± 2.48a	22.07 ± 2.49b	20.97 ± 2.46c	22.43 ± 2.46d
SD2/SD1	5.25 ± 0.42a	5.44 ± 0.43b	5.57 ± 0.38d	5.04 ± 0.39c
RQA				
*L*_max_	217.74 ± 17.39a	232.91 ± 17.43b	227.98 ± 15.42d	220.21 ± 15.46c
%DET	99.25 ± 0.17	99.29 ± 0.17	99.35 ± 0.15d	99.18 ± 0.15c
Sheep				
Time domain				
Mean HR	107.83 ± 12.79a	116.82 ± 12.79b	111.68 ± 12.59c	113.68 ± 12.59d
RMSSD	81.47 ± 1.68b	62.92 ± 1.65a	75.16 ± 1.45d	68.05 ± 1.42c
Frequency domain				
HF	36.99 ± 1.49b	33.75 ± 1.47a	31.90 ± 1.55c	37.90 ± 1.54d
LF/HF	3.00 ± 0.23a	3.73 ± 0.23b	3.69 ± 0.21d	3.17 ± 0.20c
Poincaré measures				
SD1	57.68 ± 1.19b	44.53 ± 1.17a	53.21 ± 1.02d	48.17 ± 1.00c
SD2/SD1	2.90 ± 0.16b	2.61 ± 0.16a	3.14 ± 0.15d	2.42 ± 0.15c
RQA				
*L*_max_	180.88 ± 15.89a	212.63 ± 15.82b	210.06 ± 14.23d	188.84 ± 14.15c
%DET	98.03 ± 0.34a	98.36 ± 0.33b	98.42 ± 0.32d	98.04 ± 0.31c

*^*a,b*^Without the effect of VeDBA. ^*c,d*^With VeDBA as a covariate. Values are presented as least squares mean and standard errors. Values within a row with different superscripts differ significantly at *P* < 0.05. VeDBA, natural logarithmically transformed vectorial dynamic body acceleration (*g*); HR, heart rate; HRV, heart rate variability; RMSSD, root mean square of the successive inter-beat intervals differences (ms); HF, normalized power of the high frequency band (n.u.); LF/HF, the ratio of normalized power of the low frequency band (LF) to HF; SD1, standard deviation of instantaneous HRV measured from axis 1 in Poincaré plot (ms); SD2/SD1, the ratio of standard deviation of long-term HRV measured from axis 2 of Poincaré plot to SD1; RQA, recurrence quantification analysis; *L*_*max*_:, the length of longest line of recurrent points (beats); %DET, percentage of recurrent points forming diagonal lines.*

**FIGURE 2:**
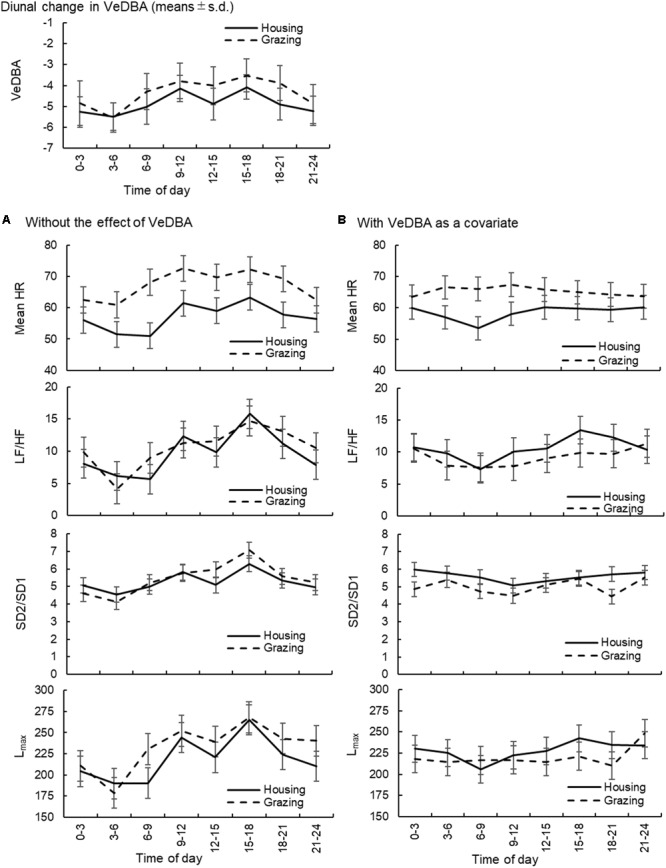
Diurnal changes in VeDBA and the effects of the change in time of day on the mean HR and HRV parameters (LF/HF, SD2/SD1, and *L*_max_) in cattle. **(A)** The changes in the mean HR and HRV parameters without considering the effect of VeDBA and **(B)** those when the effect of VeDBA was included as a covariate. The VeDBA plots are presented as arithmetic mean and standard deviations, and plots for the other parameters are presented as least squares mean and standard errors. VeDBA: natural logarithmically transformed vectorial dynamic body acceleration (*g*), HR, heart rate; HRV, heart rate variability; LF/HF, the ratio of normalized power of the low frequency band to the high frequency band; SD2/SD1, the ratio of standard deviation of long-term HRV measured from axis 2 of Poincaré plot to axis 1 in Poincaré plot; *L*_max_, the length of longest line of recurrent points (beats).

**FIGURE 3:**
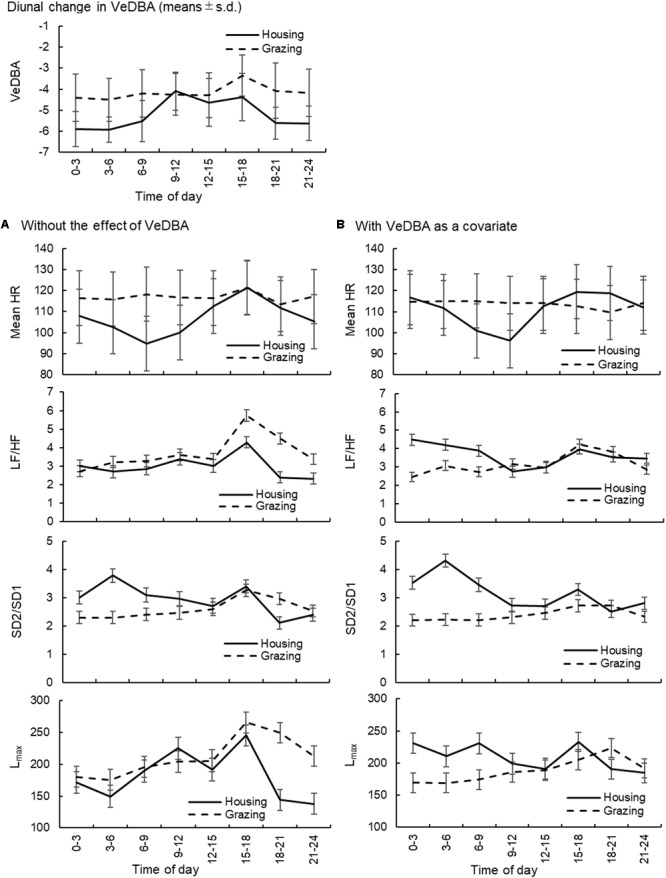
Diurnal changes in VeDBA and the effects of the change in time of day on the mean HR and HRV parameters (LF/HF, SD2/SD1, and *L*_max_) in sheep. **(A)** The changes in the mean HR and HRV parameters without considering the effect of VeDBA and **(B)** those when the effect of VeDBA was included as a covariate. The VeDBA plots are presented as arithmetic mean and standard deviations, and plots for the other parameters are presented as least squares mean and standard errors. VeDBA, natural logarithmically transformed vectorial dynamic body acceleration (*g*); HR, heart rate; HRV, heart rate variability; LF/HF, the ratio of normalized power of the low frequency band to the high frequency band; SD2/SD1, the ratio of standard deviation of long-term HRV measured from axis 2 of Poincaré plot to axis 1 in Poincaré plot; *L*_max_, the length of longest line of recurrent points (beats).

The diurnal changes in VeDBA of cattle and sheep within the two management systems are presented in **Figures 2, 3**, respectively. In both animal species, the animals managed under grazing conditions showed higher VeDBA than animals managed under housing conditions due to the higher physical activity levels of grazing animals. Moreover, the changes in VeDBA of animals within the housing system had two peaks during the daytime, which were related to the time of feeding. The effects of the change in time of day on the mean HR and the three HRV parameters (LF/HF, SD2/SD1, and *L*_max_: 1 parameter for each type of analysis) of cattle and sheep are also presented in **Figures 2, 3**, respectively. Significant interaction effects of time of day and the management systems were observed for all analyses (*P* < 0.05). In the analyses excluding the effect of VeDBA, the mean HR and *L*_max_ tended to be lower for animals managed under the housing conditions than for those managed under the grazing conditions in both animal species. However, when the effect of VeDBA was considered, *L*_max_ became higher under housing conditions than under grazing conditions (see also **Table 4**). Regarding the effect of the time of day on HRV, the diurnal fluctuations in HRV were larger, particularly for animals within the housing system, in the analyses excluding the effect of VeDBA, i.e., LF/HF and *L*_max_ were higher and HRV was thus lower during the daytime than during the nighttime for both animal species. Moreover, two significant peaks of LF/HF and *L*_max_ were observed for both animal species under housing conditions during the daytime (*P* < 0.05: statistical results are not shown in the figures), which were considered to correspond to the time of feeding. In contrast, the diurnal fluctuations in HRV, particularly for cattle, were strongly reduced in the analysis that included the effect of VeDBA, and the two peaks observed for animals within the housing system almost completely disappeared.

## Discussion

The most important finding from the present study was that the physical activity evaluated by calculating DBA from the three-dimensional acceleration data exerted significant effects on all HRV parameters studied here, as well as the mean HR. Therefore, the correction of the activity-specific component of HRV is indispensable for evaluating other effects on the HRV parameters, particularly in subjects with high physical activity levels. After excluding the effect of DBA, some of the HRV parameters showed a lower HRV in the animals within the grazing management system. However, when the effect of DBA was included, the HRV of the animals under grazing conditions became larger than that of animals managed under housing conditions, particularly when the frequency domain and RQA measures were used to evaluate HRV. By considering the effect of DBA, i.e., physical activity, we quantitatively and continuously confirmed that the stress levels of the free-moving animals within the grazing system were less than those in animals within the housing system, based on the results of the HRV analyses.

### Relationship Between HRV and Physical Activity

For healthy subjects, Hautala et al. (2010) analyzed the relationships between HRV parameters and physical activity by evaluating metabolic equivalent values (METs) estimated from one-dimensional acceleration data, and the authors reported that HR and LF/HF were positively correlated and that HF and SD1 were negatively correlated with METs in subjects with slightly higher physical activity levels. Grossman et al. (2004) also reported a systematic decrease in respiratory sinus arrhythmia (RSA), which corresponds to the HF component of HRV, as the physical activity measured using a two-dimensional accelerometer increased. As the physical activity level increases, HR increases due to a reduction in vagal modulation and concomitant sympathetic activation, while parasympathetic activity wanes and sympathetic outflow increases in a manner such that little or no vagal modulation remains (Hautala et al., 2009; Michael et al., 2017). The results of the present study were consistent with those reports, indicating that a higher physical activity level generally leads to lower HRV.

The results of the present study indicated that DBA strongly affected most of the HRV parameters and the results were substantially altered by inclusion of the effect of DBA as a covariate (**Table 4**). A robust relationship between DBA and oxygen consumption has been observed among diverse animal species (Halsey et al., 2009b, 2011; Miwa et al., 2015), and the correlation between DBA and energy expenditure is shown to be higher than correlations with other physical activity indices, such as the number of steps and raw acceleration measures (Halsey et al., 2009a; Miwa et al., 2015). The respiratory and cardiovascular systems are related by the common goal of coordinating and modulating energy expenditure and oxygen uptake to achieve optimal gas exchange and to meet changing metabolic and behavioral requirements in all vertebrates (Grossman and Taylor, 2007). Therefore, among several physical activity indices, DBA is thought to be one of the indices with the strongest correlation with HRV. As shown in **Figures 2, 3**, the inclusion of DBA as a covariate decreased the diurnal change of the HRV for both managements, indicating that DBA as a covariate could correct the diurnal changes in HRV generated by changes in daily physical activity. In particular, when HRV was evaluated for animals managed under housing conditions, two peaks in two HRV parameters (LF/HF and *L*_max_) were observed daily in the model excluding the effect of DBA, whereas the peaks became weaker when the effect of DBA was included. Thus, the change in HRV during feeding (twice for housing animals) was corrected by the use of DBA. Grossman et al. (2004) emphasized the importance of monitoring physical activity when measuring HRV and concluded that assessments considering physical activity are likely to improve the accuracy of evaluating HRV as an index of autonomic regulation in healthy and diseased subjects when regarding the context in which monitoring occurs. Therefore, the effect of physical activity, as quantified by DBA in the present study, must be corrected to evaluate the HRV of free-moving subjects.

### Effects of the Differences in Types of HRV Parameters

Currently, various types of parameters are used to evaluate HRV and are generally separated into four categories: time domain, frequency domain, geometric, and nonlinear analyses (von Borell et al., 2007). The four categories correspond to the following parameters selected in the present study: time domain, RMSSD (with mean HR); frequency domain, HF and LF/HF; geometric analysis, SD1 and SD2/SD1 in Poincaré measures; and nonlinear analysis, *L*_max_ and %DET in RQA, respectively. The responses to the inclusion of DBA as a covariate greatly differed among the categories; compared with the results obtained using the model excluding DBA, the results for the time domain parameters and Poincaré measures obtained using the model with DBA were not substantially different, but large differences were observed for the frequency domain and RQA parameters. Although the Poincaré measures are geometric parameters that can also describe nonlinear aspects of inter-beat intervals, the results were relatively similar to the results for the time domain parameters. This result was consistent with the findings from the study by Brennan et al. (2001), who reported that fitting an ellipse to the Poincaré plot does not generate indices that are independent of the standard time domain HRV indices. Although RMSSD in the time domain analysis and SD1 from the Poincaré plot are considered short-term HRV estimates, the results of the present study suggested that these parameters did not correspond to short-term changes in physical activity. In contrast, the frequency domain and RQA parameters were more susceptible to the inclusion of DBA into statistical analyses, indicating that these indices were more sensitive to physical activity and were suitable for a detailed short-term HRV analysis in free-moving subjects.

When the frequency domain and RQA parameters were compared, the results showed relatively similar trends in the present study. However, although frequency domain parameters such as LF/HF are widely used in HRV analyses, the frequency domain analysis has a serious disadvantage in analyses of HRV in freely moving animals. Notably, the quantitative analysis requires the control of respiration rate within rigid frequency ranges. Voluntary alterations in respiratory parameters influence the HF magnitude of HRV; slowing and deepening of breathing will amplify the magnitude, whereas faster, more shallow breathing may all but eliminate the magnitude (Grossman et al., 1991). Increases in breathing frequency shift the HF peak to the frequency of breathing, thereby potentially eliminating the expected HF peak and leading to false conclusions about decreased parasympathetic activity (White and Raven, 2014). Therefore, it is critical to control breathing to interpret HRV accurately (Billman, 2013b). Hence, the true frequency range of the HF component can be reliably determined only by examining the characteristics of respiration, and therefore the rigid frequency range for the HF component may be unsuitable for the correct parameterization of the HRV power spectra due to their inadequate reflection of the actual physiological condition (Cammann and Michel, 2002). Moreover, LF components are also affected by respiratory measures, which are generated from complex non-linear interactions between sympathetic and parasympathetic nerve activity (Billman, 2013b). In contrast, the nonlinear method used in the present study, i.e., RQA, requires no mathematical transformations and assumptions and only counts similar events in an embedded space (Zbilut et al., 2002). Although normal spontaneous respiratory influences might also mask the normally chaotic pattern of HRV (Fortrat et al., 1997), nonlinear dynamic techniques are based on the concept of chaos and more effectively describe the processes that are generated by complex biological systems (Acharya et al., 2006). Since many physiological processes are known to be nonlinear, the nonlinearity of HRV, rather than the linearity, is a better marker of the neurological property that subtly and appropriately responds to minor changes in environmental demands (Young and Benton, 2015). Therefore, when evaluating the HRV of free-moving subjects, the use of RQA measures corrected for physical activity by incorporating DBA into the analysis is recommended.

### DBA and HR-Corrected HRV Parameters

**FIGURE 4:**
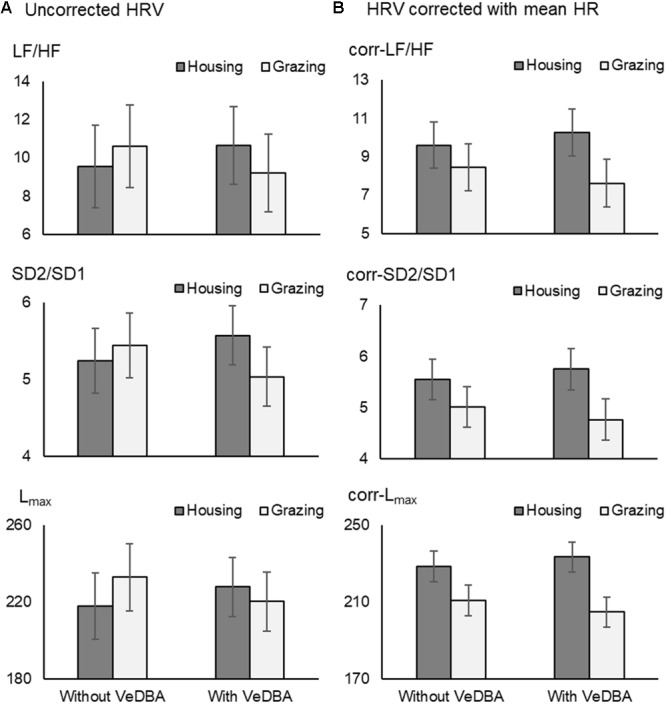
The effects of VeDBA on uncorrected HRV parameters **(A)** and HRV parameters corrected for mean HR **(B)** in cattle. The corrected HRV parameters (corr-LF/HF, corr-SD2/SD1, and corr-*L*_max_) were calculated by multiplying the uncorrected parameters by average inter-beat intervals (seconds) to the power of 2, 1, and 1, respectively. *Y*-axes in **(A)** are similar to those in **(B)**, because the average inter-beat intervals of cattle in the present study were approximately 1 s (1,000 ms). Hence, the data presented in **(A)** and **(B)** cannot be directly compared due to the differences in dimensions of the units. VeDBA, natural logarithmically transformed vectorial dynamic body acceleration (*g*); HR, heart rate; HRV, heart rate variability; LF/HF, the ratio of normalized power of the low frequency band to the high frequency band; SD2/SD1, the ratio of standard deviation of long-term HRV measured from axis 2 of Poincaré plot to axis 1 in Poincaré plot; and *L*_max_, the length of longest line of recurrent points (beats).

**FIGURE 5:**
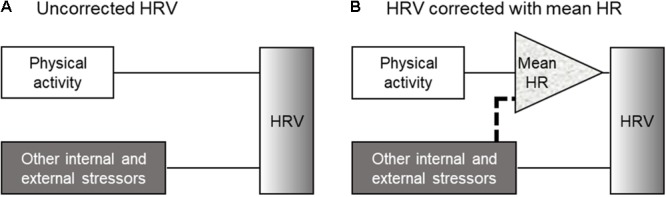
Schematic views of the analytical methods used to correct the effect of physical activity on HRV in the present study. **(A)** The inclusion of DBA as a covariate into uncorrected HRV analysis and **(B)** the inclusion of DBA as a covariate into the HR-corrected HRV analysis. When the correlation between physical activity and mean HR is extremely high, the method shown in **(A)** and the method used to correct HRV for the mean HR yielded similar results. On the other hand, when the stressors other than physical activity affect mean HR *per se*, the method shown in **(B)** could reveal the nonlinear relationship between physical activity and mean HR (*dotted line* in **B**). DBA, dynamic body acceleration; HR, heart rate; and HRV, heart rate variability.

The effect of mean HR on HRV has been recognized since Akselrod et al. (1985) explained the importance on the relationship between HR and HRV. In particularly, in recent years, several studies have tried to correct the effect of mean HR on HRV parameters (e.g., Monfredi et al., 2014; Bolea et al., 2016; van Roon et al., 2016). Among the correction methods, the strategies proposed by Sacha and Pluta (2008) and Sacha et al. (2013a,b) are thought to be simple and useful methods for analyzing HRV; if HRV parameters reveal a negative correlation with mean HR, they are divided by suitable powers of their corresponding average inter-beat intervals, and if they present a positive correlation, the correction relies on multiplication by adequate powers of average inter-beat intervals (Sacha et al., 2013a,b; Pradhapan et al., 2014; Gąsior et al., 2016). This modification method enables us to remove the HRV dependence on HR, even the physiological one, in a purely mathematical manner (Trimmel et al., 2015). To see the effect of the correction by mean HR on our results comparing HRV parameters under different management conditions, we therefore analyzed the HRV parameters with and without DBA, before and after correcting with the mean HR using the above mentioned correction method (**Figure 4**). The trend observed after correcting HRV parameters for the mean HR (**Figure 4B** “Without VeDBA”) was similar to the trend observed after including DBA as a covariate (**Figure 4A** “With VeDBA”). This result seemed to be reasonable because the correlation coefficient between physical activity and mean HR was as high as 0.7 for cattle (**Table 3**). Then, we tested the possibility that the effects of including DBA would be obscured if the HRV parameters were corrected by the mean HR before the inclusion of DBA. However, the effect of DBA was a highly significant source of variation also for the HR-corrected HRV parameters when DBA was used as a covariate in the analysis (*P* < 0.001). Moreover, the difference in the two management systems after correcting HRV parameters became more prominent by including the effect of DBA (**Figure 4B** “With VeDBA”). These results were somewhat unexpected and could be better interpreted using the block diagrams shown in **Figure 5**. In the present study, we tried to directly correct HRV components originating from physical activity using DBA as a covariate (**Figure 5A**). The results shown in **Figure 4** suggested that the mean HR used for the correction seemed to have been modulated substantially by internal and external stressors other than physical activities (**Figure 5B**). As mentioned above, in principle, physical activity inevitably increases the mean HR by enhancing physiological energy metabolism; then, the increased mean HR decreases HRV in a purely mathematical manner (Billman, 2013a; Sacha, 2013, 2014). Based on this principle, we believed that this relationship between intensity of physical activity and magnitudes of HRV should be maintained and always mediated by the net increase in the mean HR. As a result, the analysis including DBA as a covariate after correcting HRV by the mean HR could reveal the nonlinear relationship between physical activity quantified as DBA and the actual mean HR, which was most likely caused by the effects of the internal and external stressors other than physical activity on the mean HR *per se* (such as the difference in management system, ambient temperature, psychological state, modality of physical activity, the effect of allostasis as suggested by Pohlin et al., 2017, etc.). Hence, the effects of internal and external stressors on HRV mediated by the change in mean HR could be clarified by including DBA in the HR-corrected HRV analysis.

The correction of HRV parameters for mean HR is a powerful method to easily analyze data, particularly in clinical situations. The effects of various stressors other than mean HR on HRV have been assessed by weakening the dependency. In addition, by strengthening the HRV dependence on HR in the opposite way, researchers have tested whether the inherent relationships between HR and HRV are sustained and have evaluated cardiovascular risks (Sacha et al., 2013a,b; Gąsior et al., 2016). Although this method should be carefully applied to situations where the target stressors to be evaluated directly affect the mean HR, it is very simple and convenient because it does not require any additional measurements from patients. On the other hand, the method presented in this study introduces an additional measurement into the HRV analysis, which is independent of the HR measurement. Following the addition of this measurement, the present method can directly remove the confounding effect of physical activity on HRV. One of the features of the present method is that this method does not require any alteration of the units of HRV parameters, enabling us to evaluate the effect of stressors on HRV as absolute values with the units. Moreover, when the effects of other internal or external stressors, such as age and ambient temperature, on mean HR and HRV can be determined, inclusion of these effects factorially as covariates or fixed effects in the analysis in addition to DBA might help to more precisely clarify the effect of other target stressors on HRV. Although there is no general rule regarding the HR adjustments for HRV parameters (Herzig et al., 2017), the present method might also expand the range of applications of the HRV analyses in revealing the nature of free-moving animals, including humans. In conclusion, we recommend the correction of HRV parameters to clarify the effects of target stressors by selecting one or multiple adequate correction methods according to the purposes of the HRV evaluation, types of target stressors and the conditions in which HRV measurements are performed.

### Limitations and Perspectives

It should be noted that the analyses reported in the present study only included 11 individuals from two animal species and treated the effects of individual differences on the mean HR and the HRV parameters as a random effect. This strategy was employed because the present study focused on developing the methodology to consider the effect of DBA as a physical activity index on HRV and used the two animal species as examples. Although the results showed some differences between the two animal species, the effect of DBA on HRV showed a similar trend in both species. The effects of the individual differences or species on HRV would be more properly evaluated with additional individuals. Furthermore, the present study treated DBA as a linear covariate in statistical model (1), but further studies are required to determine how DBA actually affects HRV. Thus, investigations reconsidering the analytical method might be necessary in the future to improve HRV assessments under free-moving conditions.

To our knowledge, this study is the first to report an HRV analysis in which the effect of physical activity level quantified by DBA on HRV was corrected. The simplicity of acceleration loggers enables wide usage and an evaluation of DBA under several circumstances. The results indicated that activity-specific component evaluated by DBA should be considered when evaluating HRV under free-moving conditions. Although the present study was conducted using a small number of individuals, the proposed method to incorporate DBA as a quantified physical activity level into the HRV analysis is a simple way of correcting the evaluation of HRV in free-moving subjects to investigate changes in the sympatho-vagal balance related to stress or pathological conditions.

## Author Contributions

KO designed the study, collected and analyzed the data, and wrote the manuscript. YH analyzed the data, created the figures, and wrote the manuscript. MM and HA collected the data and wrote the manuscript. YY and SI collected the data. KK, HK, and HH wrote the manuscript. All authors discussed the results, contributed to manuscript revisions, and provided approval for publication.

## Conflict of Interest Statement

The authors declare that the research was conducted in the absence of any commercial or financial relationships that could be construed as a potential conflict of interest.
